# Proteomic Analysis of Resistance of Gram-Negative Bacteria to Chlorhexidine and Impacts on Susceptibility to Colistin, Antimicrobial Peptides, and Ceragenins

**DOI:** 10.3389/fmicb.2019.00210

**Published:** 2019-02-18

**Authors:** Marjan M. Hashemi, Brett S. Holden, Jordan Coburn, Maddison F. Taylor, Scott Weber, Brian Hilton, Aaron L. Zaugg, Colten McEwan, Richard Carson, Joshua L. Andersen, John C. Price, Shenglou Deng, Paul B. Savage

**Affiliations:** Department of Chemistry and Biochemistry, Brigham Young University, Provo, UT, United States

**Keywords:** chlorhexidine, ceragenins, colistine, proteomic, cross-resistance, *Pseudomonas aeruginosa*, Gram-negative bacteria

## Abstract

Use of chlorhexidine in clinical settings has led to concerns that repeated exposure of bacteria to sub-lethal doses of chlorhexidine might result in chlorhexidine resistance and cross resistance with other cationic antimicrobials including colistin, endogenous antimicrobial peptides (AMPs) and their mimics, ceragenins. We have previously shown that colistin-resistant Gram-negative bacteria remain susceptible to AMPs and ceragenins. Here, we investigated the potential for cross resistance between chlorhexidine, colistin, AMPs and ceragenins by serial exposure of standard strains of Gram-negative bacteria to chlorhexidine to generate resistant populations of organisms. Furthermore, we performed a proteomics study on the chlorhexidine-resistant strains and compared them to the wild-type strains to find the pathways by which bacteria develop resistance to chlorhexidine. Serial exposure of Gram-negative bacteria to chlorhexidine resulted in four- to eight-fold increases in minimum inhibitory concentrations (MICs). Chlorhexidine-resistant organisms showed decreased susceptibility to colistin (8- to 32-fold increases in MICs) despite not being exposed to colistin. In contrast, chlorhexidine-resistant organisms had the same MICs as the original strains when tested with representative AMPs (LL-37 and magainin I) and ceragenins (CSA-44 and CSA-131). These results imply that there may be a connection between the emergence of highly colistin-resistant Gram-negative pathogens and the prevalence of chlorhexidine usage. Yet, use of chlorhexidine may not impact innate immune defenses (e.g., AMPs) and their mimics (e.g., ceragenins). Here, we also show that chlorhexidine resistance is associated with upregulation of proteins involved in the assembly of LPS for outer membrane biogenesis and virulence factors in *Pseudomonas aeruginosa*. Additionally, resistance to chlorhexidine resulted in elevated expression levels of proteins associated with chaperones, efflux pumps, flagella and cell metabolism. This study provides a comprehensive overview of the evolutionary proteomic changes in *P. aeruginosa* following exposure to chlorhexidine and colistin. These results have important clinical implications considering the continuous application of chlorhexidine in hospitals that could influence the emergence of colistin-resistant strains.

## Introduction

**FIGURE 1:**
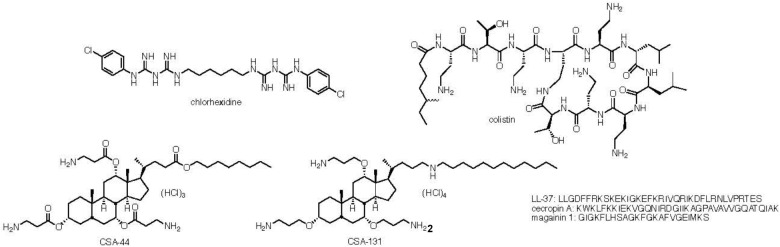
Structure of cholorhexidine, colistin and ceragenins CSA-44 and CSA-131 and sequences of LL-37, cecropin A, and magainin 1.

Chlorhexidine (Figure 1) is a potent antiseptic agent that has been in use since the 1950s (Davies et al., 1954). It is used extensively in healthcare settings in hand washing, patient bathing, treatment of gingivitis, skin antisepsis, and other antimicrobial applications (Kampf, 2016). Chlorhexidine is sparingly soluble in water, and thereby normally formulated with either acetate or gluconate to form water-soluble salts. Chlorhexidine has been found to have antibacterial, antifungal, and even some anti-viral activities (Lim and Kam, 2008). Although chlorhexidine has proven to be an effective antimicrobial agent in hospital settings, it has limitations. While data show that chlorhexidine is active against planktonic bacterial cells, it is less effective against biofilms. After treatment of *Staphylococcus aureus* (MRSA) and multi-drug resistant (MDR) *Pseudomonas aeruginosa* biofilms grown on hospital grade-surfaces, these pathogens were shown to maintain up to 11 and 80% of their cell viability respectively (Smith and Hunter, 2008). Additionally, recent studies found that various clinical isolates of *Klebsiella pneumoniae*, from before and after the widespread use of chlorhexidine, show a significant difference in susceptibility to the antimicrobial (Bock et al., 2016; Wand et al., 2017). Notably, it has been shown that development of resistance is stable; bacteria do not revert to susceptible forms after being grown for an extended time in antimicrobial-free medium, suggesting that resistance is mutational rather than adaptational (Bock et al., 2016).

Due to the prevalent use of chlorhexidine, especially in clinical settings, there has been increasing concern about whether repeated exposures of bacteria to sub-lethal doses of chlorhexidine is a factor in the emergence of MDR bacteria (Denny and Munro, 2017). For example, Palmer and coworkers found that serial exposure of vancomycin-resistant *Enterococcus faecium* to chlorhexidine resulted in reduced susceptibility to other membrane-targeting antimicrobials such as daptomycin (Bhardwaj et al., 2018). Of particular concern is the role of exposure to chlorhexidine in generation of colistin resistance. The prevalence of Gram-negative, drug-resistant bacteria has resulted in increased use of colistin (Figure 1), which is considered an antibiotic of “last resort.” However, colistin resistant organisms have been increasingly isolated from clinical settings (Wright et al., 2015; Hammerl et al., 2017). Wand et al. (2017) recently reported cross-resistance between chlorhexidine and colistin. They found that in chlorhexidine-adapted strains of *K. pneumoniae*, the minimum inhibitory concentrations (MICs) for colistin increased by a factor between 16 and 32 with five of the six strains tested. However, when colistin-resistant *K. pneumoniae* were tested for chlorhexidine susceptibility, Wand et al. (2017) found that MICs of chlorhexidine were the same with colistin-susceptible and colistin-resistant strains. On the other hand, Curiao et al. (2016) reported that at least in *Salmonella*, there are multiple mutations leading to chlorhexidine resistance that are not necessarily associated with colistin resistance.

Cross resistance of bacteria to chlorhexidine and colistin may be due to common features of these antimicrobials. For example, both are cationic (positively charged) with hydrophobic functionality. Notably, most endogenous antimicrobial peptides (AMPs, representative sequences are shown in Figure 1) also display these features: cationic groups juxtaposed with hydrophobic side chains (Hashemi et al., 2017a, 2018a). We recently reported (Hashemi et al., 2017c) that highly colistin-resistant bacteria are susceptible to both AMPs and non-peptide mimics of AMPs, termed ceragenins (representative structures are shown in Figure 1) (Hashemi et al., 2017b, 2018b,c,d). This finding led to the question of whether generation of chlorhexidine resistance results in cross resistance to ceragenins and AMPs. Reported herein are investigations of the means by which Gram-negative bacteria become resistant to chlorhexidine and how this resistance influences susceptibility to colistin, AMPs and ceragenins. Rather than focus on mutations, changes to the proteome were investigated to obtain an understanding of multiple possible alterations in gene expression that lead to resistance. We found that, as expected, induction of resistance in Gram-negative bacteria to chlorhexidine resulted in decreased susceptibility to colistin; nevertheless, organisms remained susceptible to AMPs and ceragenins. Changes to the proteome of chlorhexidine-resistant organisms, relative to a parent strain, were in the outer membrane proteins as well as proteins associated with chaperones, efflux pumps, flagella and cell metabolism.

## Materials and Methods

### Materials

*Klebsiella pneumoniae* (ATCC 13883), *Acinetobacter baumannii* (ATCC 19606), and *P. aeruginosa* (ATCC 27853) were obtained from the American Type Culture Collection (ATCC, Manassas, VA, United States). Ceragenins CSA-131 and CSA-44 were synthesized from a cholic acid scaffolding as previously described (Li et al., 1998). LL-37 (LLGDFFRKSKEKIGKEFKRIVQRIK-DFLRNLVPRTES), cecropin A (KWKLFKKIEKVGQNIRDGIIKAGPAVAVVGQATQIAK), and magainin 1 (GIGKFLHSAGKFGKAFVGEIMKS), all with > 95% purity, were purchased from Anaspec (Fremont, CA, United States) and used without further purification. Chlorhexidine, colistin (polymyxin E), congo red and coomassie brilliant blue dyes were purchased from Sigma-Aldrich (St. Louis, MO, United States).

### Susceptibility Testing

Minimum inhibitory concentrations were determined using a broth microdilution method in a 96-well plate according to the Clinical and Laboratory Standards Institute (2006). Briefly, twofold dilutions of antibacterial agents (chlorhexidine, colistin, LL-37, cecropin A, magainin 1, and ceragenins) in tryptic soy broth (TSB) for *P. aeruginosa* or Mueller Hinton (MH) for *K. pneumoniae* and *A. baumannii* were dispensed in separate wells of a 96-well plate. Aliquots (100 μL) of a prepared inoculum (10^6^ cfu/mL) were added, and plates were incubated at 37°C for 18–20 h. Negative and positive controls were included for each set of MIC measurements. The lowest concentrations of antibacterial agents that inhibited visible growth of bacteria were recorded as the MICs.

### Serial Passage of Bacteria With Chlorhexidine

Serial passaging of bacteria was performed according to a protocol described previously (Hashemi et al., 2017c). Briefly, MICs (24 h) for the strains were determined, and bacteria growing at the highest concentrations of the antimicrobials were used to inoculate fresh medium. This process was repeated every 18–24 h for 10 days. MICs were recorded every 24 h, and concentrations of the antimicrobials were adjusted to allow for determination of MICs.

### Colony Morphology Assay

MHA plates were made in 60 mm × 15 mm Petri dishes with the addition of Congo red (40 mg/L) and Coomassie brilliant blue (20 mg/L) dyes. Bacterial cultures were grown overnight in MH broth at 37°C. An aliquot of bacterial culture (1 μL) was then spotted onto the MHA dyed plates and incubated at 37°C for 24 h (Mukherjee et al., 2017). Images were acquired using a stereo microscope (Stemi 2000, Zeiss).

### Proteomic Sample Preparation

Three independent studies were performed for the proteomics analyses and one single colony was used for each independent study. Both wild-type (ATCC 27583) and drug-resistant *P. aeruginosa* were cultured in TSB (25 mL) to an OD600 nm of 0.5. Cells were pelleted at 3750 × *g* for 10 min at room temperature followed by three washes with ice-cold PBS. Cell pellets were lysed in 1% sodium dodecyl sulfate (1 mL) in Tris buffer (50 mM, pH 8). Cells were briefly sonicated to further lyse the cells. Protein concentration was determined by bicinchoninic acid (BCA) protein assay (Thermo Fisher Scientific, Waltham, MA, United States). Protein digestion was carried out using filter-assisted digestion. Aliquots of 50 μg of protein were transferred to centrifugal filters (30 kD molecular weight cutoff), denatured with guanidine (100 μL, 6 M guanidinium hydrochloride, 100 mM Tris, pH 8.5), and centrifuged for 15 min at 14000 × *g*. This step was repeated three times to remove SDS entirely. Cell lysate was reduced by DTT (20 mM in 6 M guanidinium hydrochloride) at 65°C for 5 min. After cooling for 5 min, alkylation was performed with iodoacetamide (50 mM, 1 h incubation at room temperature in the dark). The mixture was then washed two times with ammonium bicarbonate buffer (25 mM, pH 8.5). The washed, alkylated protein sample was re-suspended in ammonium bicarbonate (ABC, 100 μL) followed by overnight digestion at 37°C with trypsin (1 μg, Sigma-Aldrich Proteomics Grade). After trypsin digestion, peptides were isolated by spinning down the mixture (14,000 × *g*, 30 min) with one rinse of the filter using ABC (100 μL, centrifugation 14,000 × *g* for 30 min). The combined filtrate was vacuum dried using a speedvac (Sorval). The dried sample was immediately re-dissolved in 50 μL of buffer (0.1% formic acid in Optima-grade water) for mass spectrometry analysis (Wiśniewski et al., 2009).

### Mass Spectrometry Data Acquisition

Mass spectrometry data were collected using an Orbitrap Fusion Lumos mass spectrometer (Thermo Fisher Scientific, Waltham, MA, United States) coupled to an EASY-nLC 1200 liquid chromatography (LC) pump (Thermo Fisher Scientific, Waltham, MA, United States). A capillary RSLC column (EASY-spray column pepMap ^®^ RSLC, C18, 2 μm, 100 Å, 75 μm × 25 cm) was used for separation of peptides. The mobile phase was comprised of buffer A (0.1% formic acid in Optima water) and buffer B (Optima water and 0.1% formic acid in 80% acetonitrile).

Elution of peptides was performed at 300 nL/min with the following gradients over 2 h: 3–25% B for 80 min; 25–35% B for 20 min; 35–45% B for 8 min; 45–85% B for 2 min and 85% for 8 min. Data were acquired using the method described by Peng et al. (2017). Briefly, using the top speed method (3 s cycle) and a resolution of 120,000 at 200 m/z, a full scan MS was obtained in the orbitrap with a target value of 3e5 and a maximum injection time of 60 ms. Detection of fragment ions in the linear ion trap was performed with a target AGC value of 1e4 and a maximum injection time of 250 ms.

Label-free quantification (LFQ) values were obtained using PEAKS (version 8.5) and the Swiss-Prot validated *P. aeruginosa* proteome (5638 sequences May 2018) with a reverse sequence decoy database concatenated and included during the search. After the removal of contaminants, low scoring peptides and reverse matches, the LFQ values for the top three peptides were summed and transformed by a logarithm of 2. The proteins were then separated into wild-type and mutant groups, and only those with at least three correct LFQ values were used for quantification. Accordingly, two-tailed heteroscedastic *t*-tests were conducted to compare wild-type versus mutant groups. The acquisition of gene ontology terms and functional clusters, as well as the identification of molecular functions and biological process, was conducted using the Database for Annotation, Visualization and Integrated Discovery (DAVID) and UniProt. Information about protein-protein interaction networks was retrieved from STRING (v10).

## Results and Discussion

### Susceptibility of Tested Gram-Negative Bacteria to Antimicrobials

**FIGURE 2:**
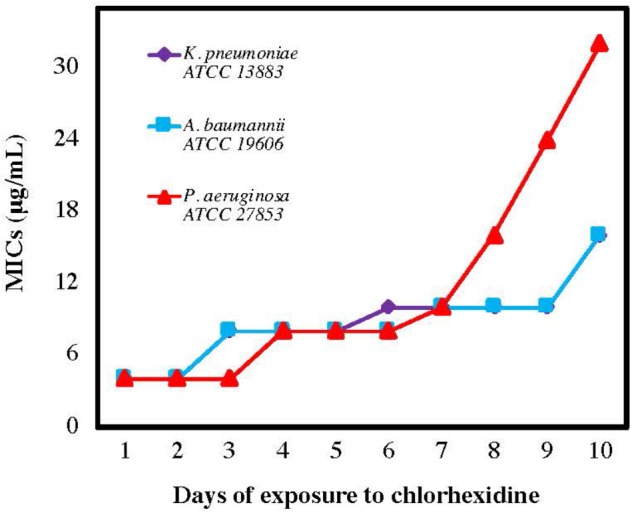
Minimum inhibitory concentrations (MICs) of chlorhexidine against *Acinetobacter baumannii*, *Pseudomonas aeruginosa*, and *Klebsiella pneumoniae* after the number of sequential exposures (24 h) to chlorhexidine.

Previous work has shown that Gram-negative bacteria become resistant to colistin following exposure to a sublethal concentration of colistin; however, colistin resistance does not correlate with higher MIC levels for AMPs or ceragenins (Hashemi et al., 2017c). To determine whether resistance to chlorhexidine also leads to cross-resistance against other antimicrobials, Gram-negative strains were serially exposed to chlorhexidine for 10 days with MIC determination every 24 h (Figure 2). MICs of AMPs (LL-37, cecropin A, magainin 1), lead ceragenins (CSA-44 and CSA-131) and colistin were measured with the chlorhexidine resistant strains (Table 1). Serial exposure of Gram-negative bacteria to chlorhexidine resulted in a rapid four- to eight-fold increase in MICs of chlorhexidine within 10 days. Notably, the chlorhexidine-resistant organisms showed decreased susceptibility to colistin (8- to 32-fold increases in MICs) in spite of not having been exposed to colistin. In contrast, MICs of representative AMPs were the same with the original strains and the chlorhexidine-resistant organisms. Similarly, the MICs of lead ceragenins (CSA-44 and CSA-131) were unchanged with the original and chlorhexidine resistant bacteria. Although chlorhexidine-resistant Gram-negative bacteria showed decreased susceptibility to colistin, as earlier reported by Wand et al. (2017) and seen in our studies, colistin-resistant Gram-negative bacteria did not show an increased MIC with chlorhexidine (data not shown). Overall, these results support the idea that exposure of Gram-negative bacteria to chlorhexidine can lead to cross-resistance to colistin, while susceptibilities to AMPs and ceragenins remain unchanged.

**Table 1:** Minimum inhibitory concentrations (MICs) of chlorhexidine, colistin, CSA-131, CSA-44, LL-37, magainin 1, and cecropin A with susceptible standard strains of *K. pneumoniae*, *A. baumannii*, and *P. aeruginosa* and with strains serially exposed to chlorhexidine and colistin.

	MICs (μg/mL)						
Strain	CHX	Col	CSA-131	CSA-44	LL-37	Magainin 1	Cecropin A
*K. pneumoniae*							
ATCC 13883	4	2	1	1	32	64	2
Serially exposed to CHX	16	16	1	1	32	64	nm
*A. baumannii*							
ATCC 19606	4	1	2	2	16	32	4
Serially exposed to CHX	16	16	2	2	16	32	nm
*P. aeruginosa*							
ATCC 27853	4	1	2	2	32	64	4
Serially exposed to CHX	32	32	2	2	32	64	nm

*CHX, chlorhexidine; Col, colistin. All tested bacteria were serially esposed to chlorhexidine for 10 passages. All the MICs are results of three independent experiments. nm, not measured.*

### Colony Morphology

**FIGURE 3:**
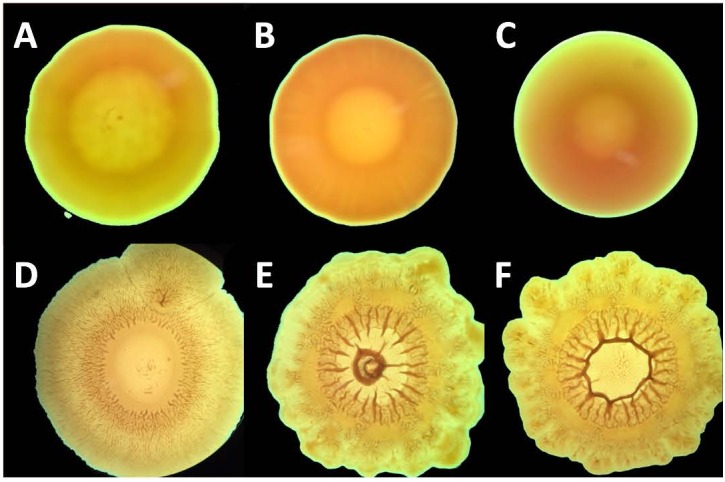
Colony morphology of *A. baumannii* and, *P. aeruginosa*, and *K. pneumoniae* upon expore to chlorhexidine. Bacterial strains *P. aeruginosa* **(A,D)**, *A. baumannii* **(B,E),** and *K. pneumoniae* **(C,F)** were serially passaged with chlorhexidine **(D–F)** and the resulting colony morphology was compared with untreated colonies **(A–C)**.

Control strains of each type of Gram-negative bacteria produced colonies of circular and smooth morphology (Figure 3A–C). The serially passaged strains of *A. baumannii* and *K. pneumoniae*, however, formed colonies of irregular shape and had rough surfaces (Figure 3D–F). Although *P. aeruginosa* serially exposed to chlorhexidine produced colonies which were circular, they also had slightly rough surfaces and formed undulating margins. All three serially passaged strains grew larger colonies that covered more surface area than their respective controls. These changes in morphology may be a response to LPS modifications. For example, in *Pseudomonas stutzeri*, it has been shown that chlorhexidine resistance accompanies a loss of low-mass LPS species in SDS-PAGE gels. Because these strains showed cross-resistance, the shift to higher-mass LPS suggests that multidrug resistance is accompanied by LPS production which favors larger polysaccharide components (Tattawasart et al., 2000). And, as demonstrated in Gram-negative *Vibrio cholerae*, colony morphology can shift from smooth to rough in response to extracellular polysaccharide changes (Limoli et al., 2015). The connection between extracellular polysaccharides and colony morphology is further supported by *Salmonella enterica*, which shifts from rough to smooth morphology when short O-antigen chain LPS production is up-regulated (Karatzas et al., 2008). Because capsular polysaccharides have been shown to shield *K. pneumoniae* from polymixins, including colistin (Formosa et al., 2015), the increased mass of the polysaccharides contained within LPS may be the bridge between resistance to the two classes of antimicrobials, either by introducing additional mass or by changing the organization of the capsule. Generally, proposed mechanisms of colistin resistance in Gram-negative bacteria involve LPS modifications (Walsh and Duffy, 2013; Hashemi et al., 2017c; Kim and Ko, 2018). Thus, chlorhexidine resistance potentially brings about colistin resistance through one or more modifications to LPS.

### Proteomics

Proteomics has been established as an effective method of analyzing biological processes and is particularly valuable for examining resistance to antibiotics (Fernández-Reyes et al., 2009). For *P. aeruginosa*, a recent study has unveiled several differences in protein expression associated with low and high levels of ciprofloxacin resistance (Peng et al., 2017). On the whole, though, proteomic data on chlorhexidine-resistant *P. aeruginosa* remain scarce, in particular regarding cross-resistance with colistin, for which, to the best of our knowledge, no data on differential expression inherent to resistance are yet available. We have evaluated cross-resistance between colistin, chlorhexidine, AMPs and their mimic, ceragenins, and we further analyzed the proteome of chlorhexidine-resistant *P. aeruginosa* using reference strain ATCC 27853 for comparison.

**Table 2:** Identification, function, and biological process of proteins differentially expressed between chlorhexidine-resistant and wild-type *P. aeruginosa*.

	Accession no.	Protein	Function	Biological process	Fold change
1	P13794	Outer membrane porin F (OprF)	Porin activity, structural role in determining cell shape and growth in low osmolarity medium.	Adhesion of symbiont to host, ion transport, regulation of cell shapes.	62.38
2	Q9I5U2	LPS-assembly protein LptD (LptD)	Assembly of lipopolysaccharide at the surface of the outer membrane.	Gram-negative-bacterium-type cell outer membrane assembly, lipopolysaccharide export, response to organic substance.	7.83
3	P50601	Tol-Pal system protein TolB (TolB)	Maintains outer membrane integrity during outer membrane invagination of cell division.	Cell cycle, cell division, protein import.	15.36
4	P50600	Tol-Pal system protein TolA (TolA)	Role in outer membrane invagination during cell division, maintains outer membrane integrity.	Bacteriocin transport, cell cycle, cell division.	285.55
5	Q9HVZ9	UDP-N-acetylmuramoylalanine—D-glutamate ligase (MurD)	Cell wall formation, catalyzes addition of glutamate to UDP-*N*-acetylmuramoyl-L-alanine (UMA).	Cell cycle, cell division, cell wall organization, peptidoglycan biosynthetic process, regulation of cell shape.	3.63
6	Q9HVD1	Lipid A deacylase PagL (PagL)	Has 3-*O*-deacylase activity, hydrolyzes ester bond at the 3 position of lipid A.	Lipid A metabolic process, lipopolysaccharide metabolic process.	Large positive
7	Q9HXY6	UDP-3-*O*-acylglucosamine *N*-acyltransferase (LpxD)	Catalyzes N-acylation of UDP-3-*O*-acylglucosamine with 3-hydroxyacyl-ACP as the acyl donor.	Lipid A biosynthetic process.	Large positive
8	P47205	UDP-3-*O*-acyl-*N*-acetylglucosamine deacetylase (LpxC)	Catalyzes hydrolysis of UDP-3-*O*-myristoyl-*N*-acetylglucosamine to UDP-3-*O*-myristoylglucosamine and acetate.	Lipid A biosynthetic process.	108.97
9	Q9I0M4	Outer-membrane lipoprotein carrier protein (LolA)	Transports lipoproteins from inner membrane to outer membrane.	Chaperone-mediated protein transport across periplasmic space, lipoprotein localization to outer membrane.	Large positive
10	P33641	Outer membrane protein assembly factor BamD (BamD)	Assembly and insertion of beta-barrel proteins to the outer membrane.	Cell envelope organization, protein insertion into membrane.	58.42
11	P11221	Major outer membrane lipoprotein (OprI)	^∗∗∗^	^∗∗∗^	973.45
12	Q9HVN5	Chaperone protein ClpB (ClpB)	Stress-induced multi-chaperone system involved in recovery from heat induced damage.	Protein metabolic process, protein refolding, response to heat.	152.40
13	Q9HV52	Protein-export membrane protein SecG (SecG)	Participates in protein export by sequence similarity.	Intracellular protein transmembrane transport, protein secretion, protein transport by the Sec complex.	Large positive
14	Q9HU56	Protein-export protein SecB (SecB)	Molecular chaperone that binds precursor proteins and maintains them in a translocation-competent state. Binds receptor SecA.	Protein tetramerization, protein transport.	Large positive
15	Q9LCT3	Protein translocase subunit SecA (SecA)	Sec protein translocase complex, couples hydrolysis of ATP to protein transfer, SecB receptor.	Protein import, protein targeting, protein transport by the Sec complex.	0.84
16	P13981	Arginine deiminase (ArcA)	Conversion of L-arginine to L-citrulline.	Arginine catabolic process to ornithine, arginine deiminase pathway.	70.59
17	P08308	Ornithine carbamoyltransferase, catabolic (ArcB)	Catalyzes phosphorolysis of citrulline in catabolism of arginine.	Arginine biosynthetic process via ornithine, arginine catabolic process to ornithine, arginine deiminase pathway, urea cycle.	650.66
18	P13982	Carbamate kinase (ArcC)	Synthesizes CO_2_ and NH_3_ from carbamoyl phosphate.	Arginine deiminase pathway, carbamoyl phosphate catabolic process.	290.09
19	P52477	Multidrug resistance protein MexA (MexA)	Periplasmic linker of MexAV-OprM efflux system, efflux pump for *n*-hexane and *p*-xylene efflux.	Protein homooligomerization, response to antibiotic.	35.89
20	Q59637	Pyruvate dehydrogenase E1 component (AceE)	Catalyzes conversion of pyruvate to acetyl-CoA and CO_2_.	Glycolytic process	3014.55
21	Q59638	Dihydrolipoyllysine-residue acetyltransferase component of pyruvate dehydrogenase complex (AceF)	Catalyzes acetyl-CoA and enzyme N_6_-(dihydrolipoyl)lysine to CoA and enzyme N_6_-(*S*-acetyldihydrolipoyl)lysine.	Glycolytic process	40.67
22	Q9HZA7	Acetyl-coenzyme A carboxylase carboxyl transferase subunit beta (AccD)	Synthesizes malonyl-Coa from acetyl-CoA	Fatty acid biosynthetic process, malonyl-CoA biosynthetic process.	129.73
23	P37799	Biotin carboxyl carrier protein of acetyl-CoA carboxylase (AccB)	Facilitates transfer of the carboxyl group to form malonyl-CoA in fatty acid biosynthesis.	Fatty acid biosynthetic process	180.37
24	Q9HZJ3	3-Ketoacyl-CoA thiolase (FadA)	Catalyzes the final step of fatty acid oxidation (in which acetyl-CoA is released).	Fatty acid beta-oxidation	Large positive
25	Q9HZP8	Enoyl-[acyl-carrier-protein] reductase [NADH] (FabV)	Catalyzes the reduction of a carbon-carbon double bond in the final reduction of the elongation cycle of fatty acid synthesis (FAS II).	Fatty acid biosynthetic process	87.20
26	O54439	Acyl carrier protein 1 (AcpP1)	Fatty acid chain carrier.	Lipid A biosynthetic process	46.21
27	P53593	Succinate—CoA ligase [ADP-forming] subunit beta (sucC)	Provides succinate binding specificity in the coupling of hydrolysis of succinyl-CoA with synthesis of ATP or GTP in TCA cycle.	Nucleoside triphosphate biosynthetic process, protein autophosphorylation, tricarboxylic acid cycle.	1522.01
28	Q51567	Succinate—CoA ligase [ADP-forming] subunit alpha (SucD)	Provides coenzyme A and phosphate binding specificity in the coupling of hydrolysis of succinyl-CoA with synthesis of ATP or GTP in TCA cycle.	Nucleoside triphosphate biosynthetic process, tricarboxylic acid cycle.	44.3522
29	Q9I587	Fumarate hydratase class II 1 (FumC1)	Catalyzes the stereospecific interconversion of fumarate to L-malate.	Fumarate metabolic process, malate metabolic process, tricarboxylic acid cycle.	68.94
30	Q9HVF1	Probable malate:quinone oxidoreductase 2 (Mqo2)	Catalyzes the redox reaction between (S)-malate and a quinone to form oxaloacetate and a reduced quinone.	Ethanol oxidation, tricarboxylic acid cycle.	923.77
31	P14165	Citrate Synthase (GltA)	Catalyzes the formation of citrate (and CoA) from oxaloacetate and acetyl-CoA.	Tricarboxylic acid cycle	316.01
32	Q9I3F5	Aconitate hydratase A (AcnA)	Catabolism of short chain fatty acids via TCA cycle, 2-methylcitrate cycle I, catalyzes isomerization of citrate to isocitrate.	Anaerobic respiration, propionate metabolic process (methylcitrate cycle), response to oxidative stress, tricarboxylic acid cycle.	40.00
33	Q9I2V5	Aconitate hydratase B (AcnB)	Catabolism of SCFAs via TCA cycle, 2-methylcitrate cycle I, isomerization of citrate to isocitrate, hydration of 2-methyl-*cis*-aconitate to (2R,3S)-2-methylisocitrate.	Propionate catabolic process (2-methylcitrate cycle), tricarboxylic acid cycle.	1161.97
34	P04739	Fimbrial protein (PilA)	Assembles type IV pili	Cell adhesion involved in single-species biofilm formation, pathogenesis, regulation of calcium-mediated signaling, single-species biofilm formation on inanimate substrate, type IV pilus-dependent motility.	105.67
35	P46384	Protein PilG (PilG)	Pilus biosynthesis and twitching motility, receives environmental signals and transduces them to pilus assembly machinery.	Phosphorelay signal transduction system	46.57
36	P43501	Protein PilH (PilH)	Member of the signal transduction system that regulates twitch motility and pilus function.	Phosphorelay signal transduction system	81.69
37	P42257	Protein PilJ (PilJ)	Member of the signal transduction system that regulates twitch motility and pilus function.	Chemotaxis	182.73
38	P34750	Fimbrial assembly protein PilQ (PilQ)	Biogenesis and secretion of type IV fimbriae, essential for formation of pili.	Protein secretion, type IV pilus biogenesis	Large positive

We used label-free quantitation to compare the relative concentrations of proteins within the proteome of wild-type and chlorhexidine-resistant *P. aeruginosa*. We observed substantial alterations of the proteome of the resistant strain relative to the wild-type strain, with several highly abundant proteins reduced in relative concentration and increases in a collection of proteins with distinct connections to antibiotic resistance as outlined below. A total of 330 proteins (out of 528 proteins) were differentially expressed at a significant level (twofold-change, *p* < 0.05), following exposure to chlorhexidine. Complete data analysis is provided as Supplementary Material. Due to the high number of proteins significantly differentially expressed, not all are described or listed in Table 2. Identification, function and biological functions of some proteins differentially expressed between the chlorhexidine-resistant and wild-type *P. aeruginosa* are listed in Table 2 in the same order that they are mentioned in the text. Large changes in protein expression indicate that the general protein concentration can vary dramatically in response to treatment (Southey-Pillig et al., 2005; Karatzas et al., 2008).

**FIGURE 4:**
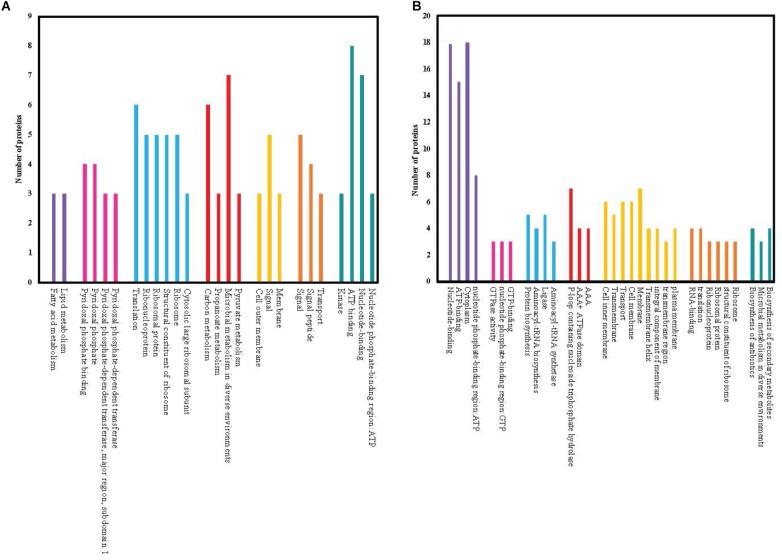
Functionally similar proteins (colored groups) among the most significantly (≥2 fold changes, *p* < 0.05) upregulated **(A)** and downregulated **(B)** proteins of chlorhexidine-resistant *P. aeruginosa* compared to wild-type. Each bar within the colored group represents a different functional connection between proteins within that group.

We searched for relationships among the most upregulated (12.5% of the total differentially expressed) and the most downregulated (12.5% of the total differentially expressed) proteins and assigned them to functional clusters. The numbers of differentially expressed proteins for each cluster are shown in Figure 4. Functional clusters containing the largest number of upregulated proteins are involved in microbial metabolism in diverse environments, carbon metabolism, ATP binding, nucleotide binding and translation of proteins. Conversely, functional clusters containing the largest number of downregulated proteins include nucleotide-binding, ATP-binding, cytoplasm and nucleotide phosphate-binding proteins.

#### Modifications in Membrane Proteins Following Exposure to Chlorhexidine

Due to the positive charge on chlorhexidine, its initial interactions with Gram-negative bacteria are thought to occur at negatively-charged regions of LPS. Further, reduced susceptibility to chlorhexidine has been previously reported in association with modifications in the cell membrane, including modifications to LPS (Tattawasart et al., 2000; Condell et al., 2014). LPS modifications that have been associated with decreased bacterial susceptibility to cationic agents, such as chlorhexidine, include charge-reducing modifications in LPS and size reduction of the O-antigen polymer (Condell et al., 2014).

In this study, several changes in the expression of proteins associated with the cell membrane were noted following chlorhexidine exposure. As shown in Table 2, OprF was one of the most highly upregulated proteins in our study. OprF is a multifunctional protein that helps maintain cell shape, interacts with peptidoglycan layers, and is associated with forming non-specific channels for passive diffusion of ions, small polar nutrients and antibiotics (Hancock and Brinkman, 2002; Li et al., 2018). It has been shown that inactivated OprF decreases virulence and increases outer membrane vesicle formation in *P. aeruginosa* (Maccarini et al., 2017). Additional work has been done on the role of OprF in modifying quorum sensing and promoting virulence factor production in *P. aeruginosa*. Loss of functioning OprF was accompanied by loss of quorum sensing network activity and virulence factor production (Fito-Boncompte et al., 2011).

**FIGURE 5:**
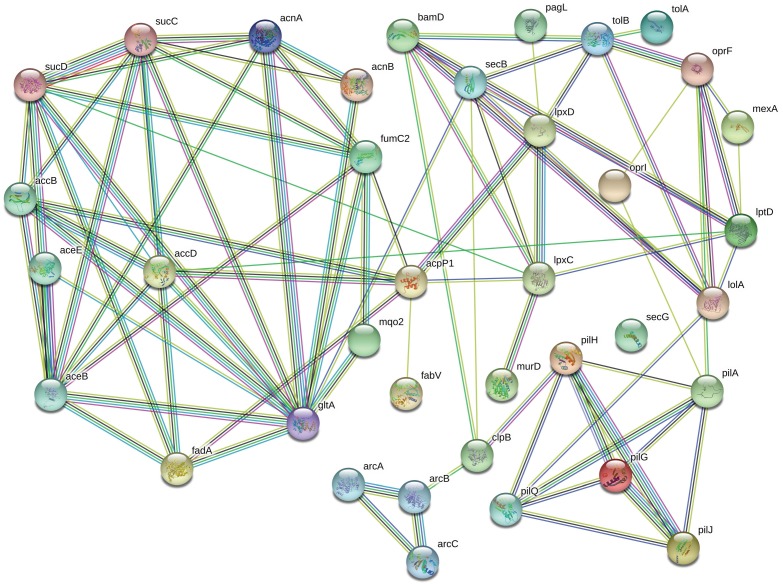
The most significantly upregulated proteins in chlorhexidine-resistant *P. aeruginosa* are highly interconnected. The connections between OprF to LptD, TolB, OprI, and LolA suggests that LPS production is substantially altered (relevant proteins are in the top right corner).

Protein network analysis (Figure 5) showed that the relative increase in OprF is accompanied by an increase in LptD concentration, a protein involved in the assembly of LPS for OM biogenesis. Mutant, non-functional LptD in *P. aeruginosa* increases the permeability of the outer membrane and results in compromised barrier function (Balibar and Grabowicz, 2015). Therefore, upregulation of LptD in our data suggests that exposure to chlorhexidine decreases the permeability of the OM in *P. aeruginosa.*

OprF also is connected to TolB (Figure 5), part of the Tol-Pal system, which contributes to the biogenesis of the OM and plays a key role in maintaining OM integrity. A significant upregulation of TolA, another part of Tol-Pal system, was also observed upon exposure of the cells to chlorhexidine in our proteomics data. Biochemical studies have identified interactions between TolA and outer membrane porins (Derouiche et al., 1995), which are consistent with our results where both TolA and OM porin F, OprF, are upregulated.

Studies have shown that mutant *tol* genes in *E. coli* increased susceptibility to antibiotics (Dennis et al., 1996). The absence of TolA in *E. coli* K-12 has been shown to cause a substantial reduction in the surface expression of O antigen (O7-specific) LPS and less lipid A-core oligosaccharide. Its absence is also correlated with attenuated growth rate and dramatic morphological changes (Gaspar et al., 2000). In *tol* mutants of *V. cholerae*, filamentation has also been reported (Heilpern and Waldor, 2000). This observation is consistent with our morphological study in which treatment with chlorhexidine caused dramatic morphological changes and altered flagella protein regulation in the Gram-negative bacteria.

An increase in the expression of a ligase enzyme associated with peptidoglycan biosynthesis (MurD) was also observed. Upregulation of MurD may result in a protective modification by increasing the thickness or cross-linking the peptidoglycan layer, which subsequently reduces the efficacy of chlorhexidine by affecting its transportation. Mur enzymes have been an attractive target for the development of antibacterial inhibitors owing to the fact that these enzymes are highly conserved molecules specific to bacteria with a known structure and function. However, no Mur inhibitors are clinically used as antimicrobials (Nikolaidis et al., 2014).

Of note, a protein which increased with chlorhexidine resistance was PagL, a deacetylase which removes the R-3-hydroxymyristate at position 3 of lipid A. PagL is activated by the *phoP/phoQ* two-component system, a regulatory system controlling genes for virulence within *Salmonella* (Olaitan et al., 2014; Park and Groisman, 2014). Though usually inhibited by 4-amino-4-deoxy-L-arabinose and phosphoethanolamine modifications of lipid A, PagL deacetylation of lipid A has been tied to increased polymyxin resistance in *Salmonella* strains lacking these modifications. In a later study, overexpression of a *pagL*-specific sRNA, Sr006, was shown to increase *pagL* mRNA, lipid A deacetylation, and polymyxin B resistance in *P. aeruginosa.* The same study also demonstrated that a *pagL* knockout resulted in decreased polymyxin B resistance (Zhang et al., 2017) Therefore, upregulation of PagL in chlorhexidine-resistant *P. aeruginosa* indicates that the mechanism of action for resistance to chlorhexidine could be partly in common with that of polymyxins.

Interestingly, protein network analysis (Figure 5) suggested that PagL is connected to LpxD, a significantly upregulated protein which is involved in lipid A biosynthesis along with LpxC, another upregulated protein. Previous MALDI-TOF analysis of the lipid A extracted from RamA-overexpressing strains of *K. pneumoniae* revealed that RamA increases colistin/polymyxin resistance levels (De Majumdar et al., 2015). This increase was accomplished through RamA directly binding to lipid A biosynthesis genes such as *lpxC* causing lipid A structural modifications. However, no observation of RamA overexpression was observed in our study. We also found several outer membrane lipoproteins including LolA, BamD, and OprI upregulated following chlorhexidine exposure. These results suggest that the increased lipoprotein synthesis of BamD and OprI might be required for appropriate sorting of lipoproteins by carrier proteins, like LolA, to the outer membrane under certain stress conditions such as antibiotic exposure (Tao et al., 2012).

#### Altered Chaperone Protein Regulation Following Exposure to Chlorhexidine

The Clp chaperones and proteases are highly conserved proteins found across the prokaryotes, in the mitochondria of eukaryotes and the chloroplasts of plants. Previous studies showed that ClpB is a chaperone associated with resistance to high temperature in *E. coli*. Furthermore, inactivation of the *clpB* gene of *Leptospira interrogans* resulted in reduced virulence and attenuated the *in vitro* growth of the cells under stress conditions, suggesting that ClpB plays a major role in bacterial survival under stress conditions (Lourdault et al., 2011). Upregulation of ClpB upon exposure to chlorhexidine was also observed in our study, confirming that ClpB is essential for stress tolerance against inducing factors like antimicrobial agents where proteins tend to unfold and aggregate. Clp protease, a periplasmic chaperone protein, was also reported to be upregulated in biofilm upon chlorhexidine treatment (Rema et al., 2016).

One of the protein translocation systems through the inner membrane involves Sec proteins in a chaperone-based pathway where immature proteins are targeted to a Sec translocase such as SecG, SecE, and SecY using the chaperone SecB for transport across the inner membrane. Our data showed upregulation of SecG and SecB while SecA was downregulated. Previous reports showed that loss of function of SecG leads to a significant increase in membrane depolarization, hydroxyl radical formation and cell death in aminoglycoside-treated cells. The redox-responsive two-component system (Arc) was also shown to have an associated role (Kohanski et al., 2008). Interestingly, our proteomics profile revealed the upregulation of ArcA, ArcB, and ArcC that encode arginine deiminase, ornithine carbamyltransferase, and carbamate kinase, respectively. The Arc two-component system contains ArcB, a quinone-sensitive sensor kinase that controls the redox state of the cellular quinone pool via modulating the phosphorylation state of the transcription factor ArcA (Kohanski et al., 2008; Liu et al., 2008).

#### Efflux Pump Activity Following Exposure to Chlorhexidine

A previous proteomics study correlated an increase in the expression of MexCD-OprJ efflux systems with a reduction in susceptibility to ciprofloxacin in *P. aeruginosa* (Peng et al., 2017). Additionally, the transcriptome of *P. aeruginosa* revealed that MexCD-OprJ efflux pump was significantly upregulated after treatment with chlorhexidine (Nde et al., 2009). Another study showed that increased expression of SmvA, an efflux pump of the major facilitator superfamily (MFS), is associated with increased resistance to chlorhexidine in *K. pneumoniae* (Wand et al., 2017). However, in our study, exposure to chlorhexidine did not increase the relative expression of SmvA or Mexcd-Oprj efflux systems. Although we did not find upregulation of SmvA or Mexcd-Opri, we found that multidrug resistance protein MexA was significantly upregulated. MexA functions as the periplasmic linker component of the MexAB-OprM efflux system that confers multidrug resistance, possibly indicating that this system plays a role in chlorhexidine response. Conversely, other research has shown that the overexpression of efflux pumps does not impact the antimicrobial efficacy of chlorhexidine, and it has been proposed that this is largely due to the limited solubility of chlorhexidine in the bacterial membrane core (Gilbert and Moore, 2005).

#### General Cell Metabolism Following Exposure to Chlorhexidine

Proteomic platform analysis showed that many proteins associated with energy metabolism and many enzymes associated with glycolysis, fatty acid biosynthesis, and the TCA cycle were upregulated in the resistant strain. For example, upregulation of AceE and AceF, two enzymes involved in the conversion of pyruvate to acetyl-CoA and CO_2_, was observed. In addition, proteins associated with acetyl-CoA carboxylase such as AccD and AccB were also upregulated. These proteins are involved in the first step of the subpathway that synthesizes malonyl-CoA from acetyl-CoA. Furthermore, significant upregulation was observed in proteins FadA, FabV and AcpP1, which are all involved in lipid metabolism. FadA catalyzes the final step of fatty acid oxidation, in which acetyl-CoA is released. FabV catalyzes the reduction of a carbon-carbon double bond in an enoyl moiety that is covalently linked to Acp as an acyl carrier protein of the growing fatty acid chain. Consistent with our results, increased expression of several proteins associated with fatty acid synthesis, such as Acp, was also observed in biofilm of *Delftia acidovorans* following chlorhexidine exposure (Kohanski et al., 2008). Finally, a substantial number of proteins associated with the TCA cycle were upregulated. Succinate-CoA ligase enzymes (SucC and SucD), fumarate hydratase (FumC1), malate:quinone oxidoreductase 2 (MqO2) and citrate synthase (GltA) were the upregulated proteins related to the first step of the cycle. Aconitate hydratase enzymes (AcnA and AcnB) were the upregulated proteins involved in isocitrate synthesis.

The acquisition of antimicrobial resistance may be energetically costly for bacteria, and in a normal environment, high-energy expenditure lowers the general fitness of bacteria. Therefore, bacteria will usually only take on metabolic costs if environmental pressures, such as antibiotics, dictate its necessity (Martínez and Rojo, 2011). The upregulation of metabolic proteins by *P. aeruginosa* shown in this study could be a result of the intrinsic linkage between bacterial metabolism and antibiotic resistance.

#### Altered Flagella Protein Regulation in Chlorhexidine Resistant Strain

Of interest in our proteomics data is the upregulation of Pil proteins (PilA, PilG, PilH, PilJ, PilQ) involved in fimbriae formation and motility. Formation of pili, or fimbriae, has been correlated with virulence in pathogens such as *P. aeruginosa*, *E. coli*, *A. baummanii*, *V. cholerae*, and *Streptococcus* spp. Presence of pili enables bacteria to bind to a surface or tissue, which initiates interaction with the host and increases biofilm formation (Tomaras et al., 2003; Fernández-Reyes et al., 2009; Martínez and Rojo, 2011). It has been previously reported that overexpression of flagellar genes is associated with increased tolerance to antimicrobial agents, including chlorhexidine, in *Salmonella*, which may now be extended to chlorhexidine tolerance in *Pseudomonas* (O’toole and Kolter, 1998; Kim and Surette, 2003; Condell et al., 2014). Twitching motility is a bacterial movement on a solid surface that occurs in a wide variety of pathogens including *P. aeruginosa*. Twitching motility is mediated by a complex chemosensory pathway including PilG, PilH, PilI, PilJ, and PilK and is also involved in biofilm formation (Whitchurch et al., 2004; Butler et al., 2010). The upregulated proteins identified in our study, including PilG, PilH and PilJ, suggest that exposure to chlorhexidine increased the twitching motility which contributes to virulence and biofilm formation. It is known that type IV pili in *P. aeruginosa* are assembled from PilA, a single protein subunit, which is transferred out of the cell via outer membrane secretin PilQ to form fimbrial strands. The upregulation of PilA and PilQ suggests that the biosynthesis of pili, which is associated with adhesion and accumulation of *P. aeruginosa* on surfaces, increased in chlorhexidine-exposed bacteria (Laverty et al., 2014). Overall, the observed alterations in Pil proteins in our comparison of the proteomes of chlorhexidine-susceptible and resistant bacteria are associated with biofilm formation and virulence, suggesting that further investigation of biofilm formation in chlorhexidine-resistant bacteria would be a meaningful line of research.

## Conclusion

We have demonstrated that prolonged exposure of Gram-negative bacteria to chlorhexidine results in significantly reduced susceptibility to colistin but not to AMPs and ceragenins. This suggests that there may be a specific mechanism for resistance of Gram-negative bacteria to chlorhexidine that causes even higher resistance to colistin but does not affect susceptibility to AMPs and ceragenins. These observations are consistent with the reported decrease in susceptibility of chlorhexidine-resistant *K. pneumoniae* to colistin (Wand et al., 2017). The fact that lead ceragenins (CSA-44 and CSA-131) retain bactericidal activity against highly colistin and chlorhexidine resistant bacteria is further support for the development of these compounds as broad-spectrum antibacterial agents to potentially be used in multiple clinical applications.

To understand the changes to Gram-negative bacteria that lead to chlorhexidine and colistin resistance but do not impact AMPs and ceragenins, an in-depth quantitative proteomic study was performed with a parent strain of *P. aeruginosa* and its chlorhexidine-resistant progeny. It is likely that proteins involved in resistance to chlorhexidine function similarly in other Gram-negative bacteria. Our study suggests that increased expression of membrane proteins such as OprF, LptD, and the Tol-Pal system is responsible for chlorhexidine resistance, but this is not the only pathway contributing to resistance. For example, chlorhexidine resistance is also associated with upregulation of PagL, which is affected by the two-component regulatory system *phoP/phoQ*. Additionally, upregulation of flagella proteins, chaperones and proteins associated with energy metabolism were also observed in the chlorhexidine-resistance pathway. Upregulation of some of these proteins also correlates with colistin resistance. Co-resistance with colistin suggests that LPS modification along with changes in expression of associated membrane proteins could be involved in the primary mechanism of chlorhexidine resistance. The fact that exposure of Gram-negative bacteria to chlorhexidine may lead to colistin resistance and possible drug resistance among Gram-positive organisms suggests that wide-spread use of chlorhexidine should be reconsidered. In order to decrease the speculated effect of chlorhexidine on the development of colistin-resistant strains, application of other antimicrobials such as ceragenins could be a safe choice considering its lower risk of cross-resistance.

## Author Contributions

MH and PS designed the experiments and wrote the manuscript. BSH, JC, MT, SW, BH, AZ, CM, RC, and SD performed the experiments. MH, JA, and JP performed data analysis.

## Conflict of Interest Statement

PS is a paid consultant for N8 Medical and CSA Biotech. The remaining authors declare that the research was conducted in the absence of any commercial or financial relationships that could be construed as a potential conflict of interest.

## Supplementary Material

TABLE S1 Proteomic data analysis: peptides and proteins.
